# Human cholesteryl ester transfer protein lacks lipopolysaccharide transfer activity, but worsens inflammation and sepsis outcomes in mice

**DOI:** 10.1194/jlr.RA120000704

**Published:** 2020-12-15

**Authors:** Aloïs Dusuel, Valérie Deckert, Jean-Paul Pais de Barros, Kevin van Dongen, Hélène Choubley, Émilie Charron, Naig Le Guern, Jérôme Labbé, Stéphane Mandard, Jacques Grober, Laurent Lagrost, Thomas Gautier

**Affiliations:** 1 INSERM/University of Bourgogne Franche-Comté LNC UMR1231 and LipSTIC LabEx, UFR Sciences de Santé, Dijon, France; 2 University Hospital of Dijon, Dijon, France

**Keywords:** cholesterol, infection, inflammation, lipoproteins, plasma lipid transfer proteins, endotoxins, lipopolysaccharide (LPS), sterol metabolism, sepsis, bacteremia, ALT, alanine aminotransferase, BPI, bactericidal/permeability increasing protein, CD14, cluster of differentiation 14, CE, cholesteryl ester, CETP, cholesteryl ester transfer protein, CETPTg mice, transgenic mice expressing human CETP, CLP, cecal ligation and puncture, IL-10, interleukin 10, IL-1β, interleukin 1 beta, IL-6, interleukin 6, LBP, lipopolysaccharide binding protein, LCMS2, liquid chromatography coupled with tandem mass spectrometry, LpB, apolipoprotein B-containing lipoproteins, LPS, lipopolysaccharide, LT/LBP, lipid transfer/lipopolysaccharide binding protein, MCP-1, monocyte chemoattractant protein 1, MIP-2, macrophage inflammatory protein 2, PLTP, phospholipid transfer protein, PLTP KO mice, PLTP knocked-out mice, PMNL, polymorphonuclear Leukocyte, RLT, reverse lipopolysaccharide transport, SIRS, systemic inflammatory response syndrome, TLR-4, toll-Like receptor 4, WBC, white blood cell

## Abstract

Bacterial lipopolysaccharides (LPSs or endotoxins) can bind most proteins of the lipid transfer/LPS-binding protein (LT/LBP) family in host organisms. The LPS-bound LT/LBP proteins then trigger either an LPS-induced proinflammatory cascade or LPS binding to lipoproteins that are involved in endotoxin inactivation and detoxification. Cholesteryl ester transfer protein (CETP) is an LT/LBP member, but its impact on LPS metabolism and sepsis outcome is unclear. Here, we performed fluorescent LPS transfer assays to assess the ability of CETP to bind and transfer LPS. The effects of intravenous (iv) infusion of purified LPS or polymicrobial infection (cecal ligation and puncture [CLP]) were compared in transgenic mice expressing human CETP and wild-type mice naturally having no CETP activity. CETP displayed no LPS transfer activity in vitro, but it tended to reduce biliary excretion of LPS in vivo. The CETP expression in mice was associated with significantly lower basal plasma lipid levels and with higher mortality rates in both models of endotoxemia and sepsis. Furthermore, CETPTg plasma modified cytokine production of macrophages in vitro. In conclusion, despite having no direct LPS binding and transfer property, human CETP worsens sepsis outcomes in mice by altering the protective effects of plasma lipoproteins against endotoxemia, inflammation, and infection.

Sepsis is a life-threatening condition characterized by a suspected or proven infection followed by a systemic inflammatory response syndrome (SIRS). The dysregulated inflammatory response of the host can lead to multiple organ failure (i.e., the main feature of severe sepsis), which may cumulate with persistent hypotension in the most advanced stage of septic shock. In 2011, sepsis was the most expensive condition treated in the United States, with annual hospital costs exceeding $20 billion (1). Even if related infection cannot be identified in a systematic way in the hospital, bacteremia is one major and leading trait of sepsis. Gram-negative bacteria, which are identified in 62% of cases (2), can release lipopolysaccharides (LPS) in bloodstream. LPS, or endotoxins, are amphipathic molecules with proinflammatory properties. They activate TLR-4/CD14 complex in leukocytes, leading to cytokine release and SIRS (3).

Importantly, the bulk of LPS cannot be metabolized in the body, with the exception of some deacylation reactions that may occur (4). To get rid of LPS, mammal body relies on one main pathway through which LPS can be disaggregated, transported in plasma, and excreted in the bile. This pathway, known as the reverse LPS transport (RLT) pathway, consists of three major steps: *1*) LPS disaggregation and binding to circulating lipoproteins, *2*) uptake of lipoprotein-bound LPS by the liver, and *3*) LPS excretion in the bile (5). Importantly, whereas lipoproteins are recognized as the main carriers of LPS in plasma, RLT involves key proteins that belong to the lipid transfer/LPS binding protein (LT/LBP) gene family and play differential roles in LPS metabolism. It includes LBP, BPI, PLTP, and CETP.

LBP is an acute phase protein that disaggregates, binds, and presents LPS to the TLR-4/CD14 complex and contributes to monocyte activation (6). Bactericidal/permeability-increasing protein (BPI) is stored in primary granules of polymorphonuclear leukocytes. BPI inhibits bacterial growth by binding LPS and forming complexes directly to the outer membrane of bacteria, thus preventing leukocyte activation (7, 8). In human and animal studies, recombinant BPI was found to be relevant in binding and neutralizing LPS (9). Unlike LBP and BPI, phospholipid transfer protein (PLTP) has been initially mainly studied in the context of lipid transport, atherosclerosis, and cardiovascular diseases. Importantly, however, PLTP was lately reported as a major component of RLT. Among LPS detoxifying properties is its ability to bind and transfer LPS between lipoproteins (10), to foster LPS excretion in the bile (5), and to kill bacteria (11). Finally, cholesteryl ester transfer protein (CETP) was mostly studied in the context of cholesterol metabolism and atherosclerosis because of its ability to exchange cholesteryl esters (CEs) and triglycerides (TGs) between high density lipoproteins (HDLs), low density lipoproteins (LDLs), and very low density lipoproteins (VLDLs). However, and unlike LBP, BPI, and PLTP, which are today well recognized as LPS binding proteins, the role of CETP in LPS metabolism remains puzzling.

In transgenic mice expressing human CETP under the control of its natural flanking regions, LPS administration leads to a decrease in plasma CETP activity and liver CETP mRNA levels (12). In patients with sepsis, a pronounced reduction of CETP plasma concentration on the first day of admission was associated with a higher mortality rate (13). Although these earlier data suggest that CETP might play a role in LPS transport and metabolism, it is not known whether altered CETP levels in patients is a cause or a consequence or sepsis severity. In addition, it remains unclear at this stage whether CETP can actually modulate inflammation and sepsis in vivo and whether it might be involved in LPS transport either directly through its ability to bind LPS with a very low affinity (14) or indirectly through its ability to modify lipoprotein structure, composition, and kinetics (15).

In the present study, we investigated whether CETP may contribute to binding and transfer of LPS in plasma, in particular in comparison with PLTP as a main LPS transfer protein in the bloodstream. To this end, we set up in vitro methods to measure LPS transfer activity between lipoproteins. In parallel, the contribution of CETP to resistance against sepsis and its harmful consequences was investigated by comparing wild-type (WT) and heterozygous human CETP-expressing (CETPTg) mice in two models of acute inflammation, i.e., after intravenous (iv) injection with purified LPS or induction of polymicrobial infection as obtained after cecal ligation and puncture (CLP). Here, concentration of LPS in the plasma, liver, and bile compartments could be monitored for the first time using a direct mass assay of LPS.

## Material and methods

### LPS transfer activity

#### Preparation of donor sepharose-CNBr-HDL-LPS-DOTAGA-Bodipy-NCS complex

##### Conjugation of DOTAGA-Bodipy-NCS to LPS

DOTAGA-Bodipy-NCS was obtained as previously described (16). LPS from *Salmonella enterica* serotype Minnesota (Sigma-Aldrich) was resuspended in sodium bicarbonate buffer at a concentration of 10.0 g/L. One equivalent of DOTAGA-Bodipy-NCS was suspended in DMSO at a concentration of 10.0 g/L and was incubated with LPS for 1 h at 37°C and 500 rpm shaking. Labeled LPS was separated from free DOTAGA-Bodipy-NCS by liquid chromatography using a Superdex 75 column (GE Healthcare) and dialyzed against PBS.

##### Conjugation of CNBr-activated sepharose to HDL

HDLs were isolated from human plasma provided by the French Blood Establishment (EFS). CNBr-activated sepharose 4B (GE Healthcare, 0.86 g) was suspended in 3.0 mL of 1.0 mM HCl buffer. The gel was then washed with coupling buffer (NaHCO_3_ 0.2 M, NaCl 0.5 M, pH 8.3) and incubated in the presence of human HDL (27.0 mg protein) overnight at 4°C. During incubation, the tubes were continuously inverted to avoid sedimentation. Excess of ligand was washed with coupling buffer, and unbound sites were blocked with Tris-HCl buffer (0.1 M). The gel was then washed with acetate buffer (0.1 M) and coupling buffer three times consecutively, before being equilibrated with PBS.

##### Conjugation of CNBr-activated sepharose to VLDL

VLDLs (7.15 mg protein) were isolated from human plasma and coupled to CNBr-activated sepharose 4B (0.28 g) as described above for HDL.

##### Binding of LPS-DOTAGA-Bodipy to Sepharose-CNBr-Lipoproteins

Sepharose-CNBr-HDL or Sepharose-CNBr-VLDL was incubated with LPS-DOTAGA-Bodipy for 1 h at 37°C with continuous inversion (1.0 mg of LPS for 2.0 mg of HDL protein or 3.6 mg VLDL protein). Tubes were centrifuged (1 min, 14,000 *g*), and pellets were washed with PBS three times consecutively to discard unbound LPS-DOTAGA-Bodipy-NCS.

#### Incubation of sepharose-CNBr-lipoprotein-LPS-DOTAGA-Bodipy with plasma

Donor Sepharose-HDL (20 μg protein) or Sepharose-VLDLs (16.8 μg protein) with bound LPS-DOTAGA-Bodipy were incubated in the presence of 16 μl (≈0.55 mg of total protein) of plasma from WT, CETPTg, or PLTPKO mice as a source of endogenous acceptor lipoproteins and lipid transfer proteins in a final volume of 100 μl PBS. The mixtures were incubated at 37°C under continuous inversion. At indicated time point, tubes were centrifuged during 1 min at 14,000 *g* to pellet donor lipoprotein complex. Fluorescence of transferred LPS-DOTAGA-Bodipy was measured in supernatant with a Wallac Victor^3^ (ThermoFisher) plate reader (excitation 485 nm, emission 535 nm).

### Animals

The experimental protocol was approved by the University of Burgundy's Ethics Committee on the Use of Laboratory Animals. PLTP-deficient (PLTPKO) mice (17) and heterozygous transgenic mice expressing human CETP (CETPTg mice) (18) were bred on a homogeneous C57BL/6J genetic background. We used male CETPTg, PLTP KO, and wild-type (WT) C57BL/6J control mice aged from 8 to 16 weeks. Animals had unlimited access to water and standard chow diet (A03-10, Safe) and were housed under a 12 h day-night cycle.

### Blood and organ sampling

Blood was collected with heparin via retroorbital puncture for intermediate time points and via cardiac puncture for final time points. Plasma was isolated by centrifugation (10 min at 6,000 *g*). Plasma lipoproteins were isolated by sequential ultracentrifugation (Apolipoprotein B-containing lipoproteins (LpB): d < 1.063; high density lipoproteins (HDL): 1.063 < d < 1.210). Liver was collected after cardiac puncture and sacrifice and immediately placed in liquid nitrogen before storage at −80°C.

### Injection of purified LPS

To assess the kinetics of LPS clearance and inflammation, a first set of WT and CETPTg mice were injected intravenously (iv) with a single dose (1.0 mg / kg body weight) of purified LPS (*Escherichia coli* O55:B5, Sigma Aldrich). Blood was sampled before (0 min) and 30 min, 1, 3, 6, 24 h after LPS injection on each animal. A second set of WT and CETPTg mice was injected iv with a single dose (15 mg/kg body weight) of purified LPS (*E. coli* O55:B5, Sigma Aldrich) and monitored during 10 days to assess survival. In experiments assessing cytokine burst as well as alterations in plasma lipoprotein profile and leukocyte counts in response to acute endotoxemia, samplings were conducted at 0 and 2 h after LPS injection.

### Cecal ligation and puncture (CLP)

Mice were anesthetized with isoflurane (Vetflurane, Virbac) and placed on a heating plate during surgery. For each animal, abdomen was shaved and disinfected with ethanol and sterile gauzes before performing midline laparotomy. The cecum was *1*) exteriorized, *2*) ligated at 75% from its distal end using 4-0 surgical suture, *3*) perforated through-and-through with a 21-gauge needle to *4*) extrude a small amount of luminal content. The surgical procedure for sham animals only consisted of step 1): After gently replacing the cecum in the peritoneal cavity, abdominal muscle and skin were sutured using 6-0 surgical suture and wound clips, respectively. Mice were injected subcutaneously with 0.4 mL of saline to compensate hydric loss that occurred during the procedure and awakened in warm conditions using an infrared bulb during 1 h. After CLP procedure, WT and CETPTg mice were monitored during 10 days (four times in the course of daytime) to assess survival. A second set of WT and CETPTg mice was used to sample blood before CLP (0 h) and sacrificed 6 or 24 h after CLP to sample blood, bile, and organs.

### CETP activity

CETP activity was measured in the plasma with commercial assay kit (Roar Biomedical) according to the manufacturer's instructions. Briefly, the plasma was incubated with quenched donor particles and acceptor particles at 37°C. The increase of fluorescence of the reactive mix is related to CETP activity. CE transfer activity was calculated from the slope of fluorescence increase between 0 and 30 min of incubation. Blank values obtained from WT plasma were subtracted from values obtained in CETPTg plasma.

### Plasma parameters

Total cholesterol, free cholesterol, TGs, phospholipids, glycaemia, and alanine transaminase activity (ALT) were measured in the plasma and/or lipoprotein fractions using commercially available enzymatic kits on an Indiko automated system (ThermoFisher). Esterified cholesterol levels were calculated by subtracting free cholesterol from total cholesterol values.

### FPLC analysis of plasma lipoproteins

Pooled plasma samples (200 μl, seven mice per group) were injected on a Superose 6 HR 10/30 column (GE Healthcare) that was connected to a fast protein liquid chromatography system (Amersham Biosciences). Lipoproteins were eluted at a constant 0.3 mL/min flow rate with 50 mM Tris-buffered saline containing 1 mM EDTA and 0.02% sodium azide. Cholesterol, phospholipid, and protein concentrations were assayed in individual, 0.3-mL fractions. VLDL, LDL, and HDL were contained in fractions 7–11, 12–22, and 23–36, respectively.

### Cytokine and chemokine assay

Concentrations of interleukin 1 β (IL-1β), interleukin 6 (IL-6), interleukin 10 (IL-10), interferon γ (IFNγ), tumor necrosis factor α (TNFα) macrophage chemoattractant protein 1 (MCP-1) and macrophage inflammatory protein 2 (MIP-2) in plasma or culture media were determined using a Milliplex MAP Mouse Cytokine/Chemokine Magnetic Bead Panel kit (EMD Millipore), according to the manufacturer’s instructions. Samples were analyzed with a Bio-Plex 200 device (Bio-Rad) using Luminex xMAP technology.

### Isolation and treatment of bone-marrow-derived macrophages (BMDM)

Bone-marrow-derived macrophages (BMDMs) were harvested from CETPTg and WT mice. Rear leg bones (tibia and femur) were flushed using PBS containing 2 mM EDTA and 1% penicillin-streptomycin and filtered using a 70 μm cell strainer. Red blood cells were lysed (NH_4_Cl 150 mM, KHCO_3_ 1 m M, EDTA 100 mM) before culture in endotoxin-free RPMI 1640 with L-glutamine containing 10% fetal bovine serum (FBS), 1% penicillin-streptomycin, and 50 ng/mL macrophage colony-stimulating factor (M-CSF, Miltenyi Biotec). Cells were plated at 4.0 × 10^5^ cells/mL in 12-well plates (5% CO_2_, 37°C) during 7 days, with medium replaced every 48 h. After differentiation, cells were treated during 2, 6, or 24 h with 10, 100, or 1,000 ng/mL LPS (Escherichia coli O55:B5, Sigma Aldrich) in RPMI 1640 with L-glutamine supplemented with 10% pooled plasma from CETPTg or WT mice.

### RNA isolation and quantitative PCR

RNAs were isolated from liver homogenates with TRIzol Reagent (Life Technologies) followed by chloroform and isopropanol extraction and quantified with a NanoDrop system (Thermo Fisher Scientific). Five-hundred nanograms of RNA was reverse-transcribed into cDNA using M-MLV reverse transcriptase, random primers, and RNAseOUT inhibitor (Invitrogen). RNAs were isolated from cells using RNeasy® Mini Kit (Qiagen) and retrotranscripted with High-Capacity cDNA Reverse Transcription Kit (Applied Biosystems) according to the manufacturers' instructions. Quantitative PCR was performed using StepOne Plus™ Real-Time PCR System (Applied Biosystems) with TaqMan Universal Master Mix II (Thermo Fisher) and the following TaqMan probes (Applied Biosystems): *Il1beta*, Mm00434228_m1; *Il6*, Mm00446190_m1; *Il10*, Mm01288386_m1; *Mcp1*, Mm00441242_m1; *Tnfalpha* Mm00443258_m1. Housekeeping gene was *Rplp0*, Mm00725448_s1. All results were expressed as fold increase compared with untreated condition (no LPS).

### Leukocyte counts in biological samples

Fresh blood samples were drawn from retroorbital plexus (10 μL) at baseline and 2 h after LPS injection. At indicated times after CLP, mice were euthanized and submitted to peritoneal lavage with 5 mL PBS. Samples were immediately analyzed on a “Scil Vet abc Plus+” automatic device (scil Animal Care, Altorf, France) in order to obtain total leukocyte (WBC, white blood cells) lymphocyte, monocyte, and polymorphonuclear leukocyte (PMNL) counts.

### LPS mass quantitation

LPS concentration in samples was determined by direct quantitation of 3β-hydroxymyristate, a major component of the Lipid A moiety, by liquid chromatography coupled with tandem mass spectrometry (LCMS^2^) as previously described (19). Briefly, an internal standard (3β-hydroxytridecanoate) was added to samples before hydrolysis with HCl for 4 h at 90°C. The resulting free fatty acids were extracted with a hexane/ethyl acetate solution and redissolved in absolute ethanol after vacuum evaporation. Fatty acids were separated by HPLC using a Poroshell 120 SB-C18 column and a 1260 Infinity LC system (Agilent Technologies) and quantitated with a 6490 triple quadrupole mass spectrometer (Agilent Technologies).

### Statistical analysis

Data are presented as means ± SD and were analysed using GraphPad Prism v6.01 (GraphPad Software Inc). Wilcoxon rank test was used to compare matched experimental groups within repeated, independent experiments for kinetics of LPS transfer activity in vitro. Survival rates were compared using Kaplan-Meier method, and the statistical significance was determined by log-rank test or χ^2^ test for specific time points. Correlations were analyzed using nonparametric Spearman test. The other experiments were analyzed using a nonparametric Mann-Whitney test for pairwise comparison. When more than two conditions were considered, nonparametric Kruskal-Wallis test followed by Dunn's test was used for multiple comparisons. All statistical analyses are considered as significant with a *P*-value ≤ 0.05.

## Results

### CETP does not transfer LPS in vitro

**Fig. 1:**
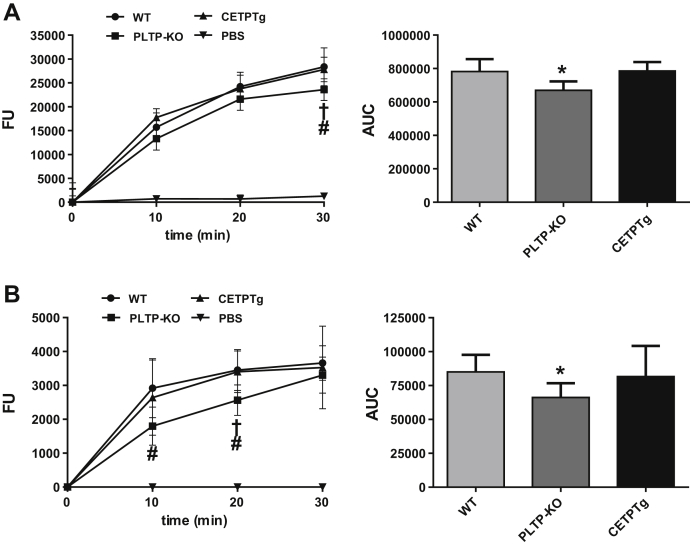
Role of CETP in LPS transfer activity between lipoproteins. DOTAGA-Bodipy-LPS transfer activity was measured between human lipoproteins and acceptor plasma from WT, PLTP KO, and CETPTg mice (n = 6 to 8). Time course of fluorescence increase reflecting the amounts of LPS transferred from human donor HDL (A) or VLDL (B) to plasma after 0, 10, 20, and 30 min of incubation at 37°C. Subsequent AUC was calculated between 0 and 30 min of incubation (right panels). Data are means ± SD. †*P* < 0.05 PLTP-KO versus WT, #*P* < 0.05 PLTP-KO versus CETPTg; Wilcoxon test. ∗*P* < 0.05 PLTP-KO versus WT; Kruskal-Wallis followed by Dunn's multiple comparisons test. AUC, area under curve; FU, fluorescence units.

We set up specific fluorescent assays for quantitation of LPS transfer activity. To this end, purified LPS from *Salmonella* Minnesota, labeled with a probe bearing a BODIPY dye, was associated with HDL or VLDL donors, and the course of its transfer toward plasma lipoprotein acceptors was monitored over a 30-min incubation at 37°C. As shown in Fig. 1, labeled LPS transferred from HDL (Fig. 1A) or from VLDL (Fig. 1B) toward endogenous lipoproteins in a time-dependent manner in plasma of wild-type mice, whereas no significant transfer occurred when donor lipoproteins were incubated alone. The amount of LPS transferred from HDL progressively increased over the 30-min period (Fig. 1A). With VLDL, and for the highest transfer rates, the transfer reached a plateau value between 20 and 30 min of incubation (Fig. 1B). As expected from earlier studies (5), the overall transfer of LPS from HDL in PLTP-KO plasma was 19% lower than that in WT plasma after 30 min resulting in a 14.6% reduction in AUC of total transfer compared with WT (*P* < 0.05). Initial LPS transfer values from VLDL were significantly lower in PLTP-KO mice, although reaching a similar level after 30 min. This resulted in a 26.7% reduction in the AUC of total transfer over 30 min compared with WT mice (*P* < 0.05). In accordance with earlier reports, it indicates that plasma PLTP significantly contributes to LPS binding and transfer between lipoproteins in plasma. Importantly, expression of human CETP in CETPTg mice did not modify plasma LPS transfer rates, which did not differ from those measured in WT mice with naturally no CETP expression (Fig. 1A, B). It indicates that CETP, in contrast to the related PLTP, is unlikely to play a significant and direct role in the binding and exchange of LPS between plasma lipoproteins.

### The presence of CETP in mice alters the kinetics of purified LPS and its biological effects in vivo

**Fig. 2:**
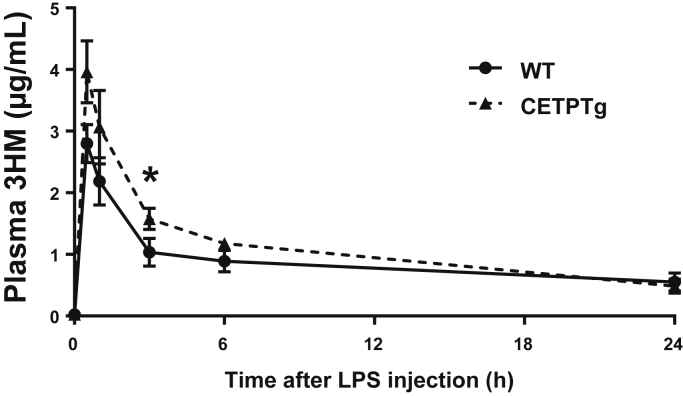
Plasma 3-HM concentration after LPS injection. WT (n = 4) and CETPTg (n = 4) mice were iv injected with LPS (1.0 mg/kg BW). LPS concentration in plasma was monitored at 0 min, 30 min, 1 h, 3 h, 6 h, and 24 h by direct 3-hydroxymyristate (3-HM) quantitation using tandem mass spectroscopy. Data are means ± SD. ∗ *P* < 0.05 WT versus CETPTg; Mann-Whitney test.

In order to determine whether CETP can alter the clearance of endotoxins in vivo, and the consequences on cytokine production, purified LPS was iv injected in WT and CETPTg mice. LPS concentration in the plasma was monitored by using a direct mass assay using the LCMS_2_ method previously described (19). We could observe a faster catabolic rate of LPS in WT than in CETPTg mice with significantly lower LPS levels 3 h after injection (1.04 ± 0.27 versus 1.58±0.08 μg/mL, respectively, *P* < 0.05, Mann-Whitney) suggesting that detoxification of LPS is slower when CETP is expressed (Fig. 2).

**Fig. 3:**
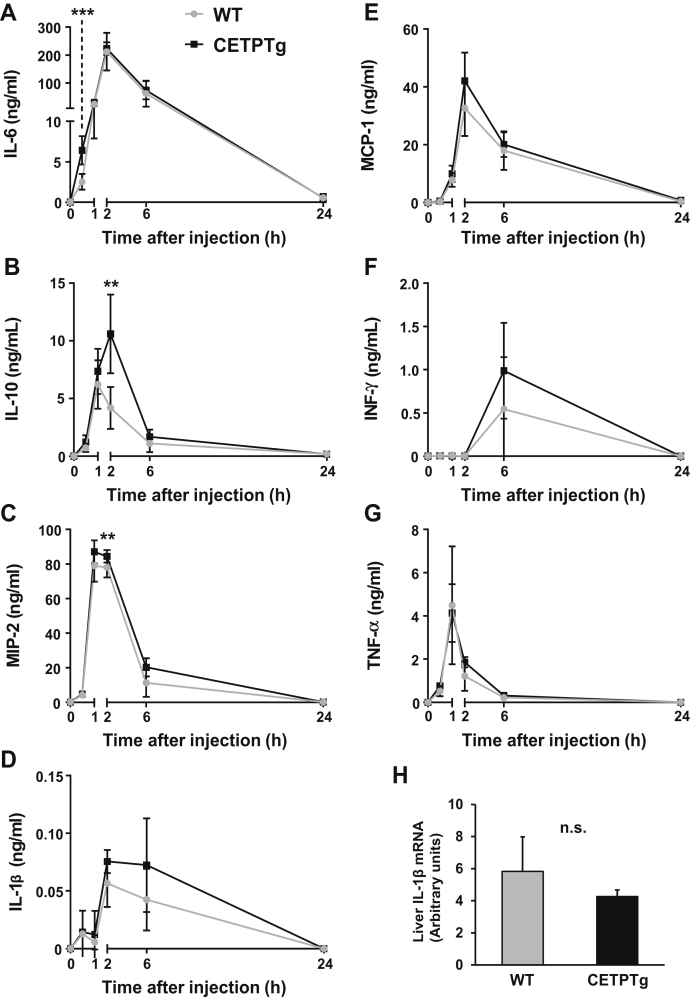
Cytokine production after LPS injection. WT (n = 8) and CETPTg (n = 7) mice were iv injected with purified LPS from E. coli O55:B5 (1.0 mg/kg BW). Plasma levels of IL-6 (A), IL-10 (B), MIP-2 (C), IL-1β (D), MCP-1 (E), IFNγ (F) and TNFα (G) were measured using Luminex assay. Liver mRNA levels of IL-1β were determined 24 h after LPS injection. Data are means ± SD. ∗∗*P* < 0.01, ∗∗∗*P* < 0.001, WT versus CETPTg; Mann-Whitney test. n.s., nonsignificant.

**Fig. 4:**
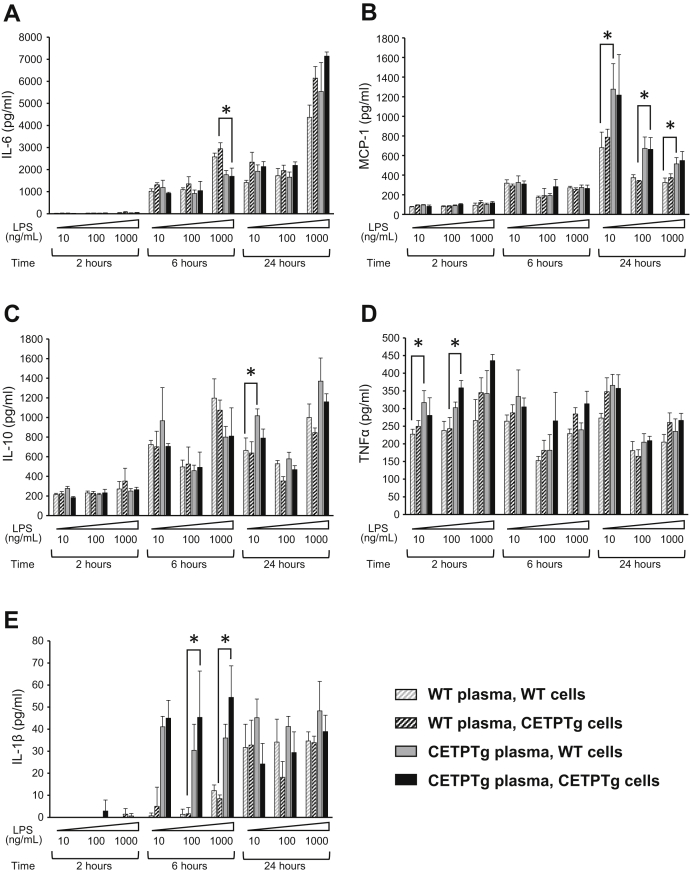
Cytokine release in the presence of plasma and BMDM from WT and CETPTg mice . Bone-marrow-derived macrophages (BMDM) were isolated and pooled from WT (gray, n = 9) or CETPTg mice (black, n = 9) and cultured as described under Materials and Methods. Differentiated cells were treated in the presence of increasing amounts of LPS (10, 100, or 1,000 ng/mL) in the presence of 10% serum from either WT (n = 9, hatched bars) or CETPTg mice (n = 8, filled bars) for 2, 6, or 24 h. BMDM response to LPS was assessed by measuring levels of secreted IL-6 (A), MCP-1 (B), IL-10 (C), TNFα (D), and IL-1β in the supernatants by multiplex assay. Data are means ± SD of three different wells and were compared by using Kruskal-Wallis followed by Dunn's multiple comparisons test. ∗ *P* < 0.05.

We observed a trend toward a more pronounced rise of plasma IL-6 (6.40 ± 1.75 versus 2.52 ± 0.97 ng/mL) (Fig. 3A, 30 min), IL-10 (10.61 ± 3.42 versus 4.19 ± 1.83 ng/mL) (Fig. 3B, 2 h), and MIP-2 (84.49 ± 3.63 versus 78.05 ± 5.76 ng/mL) (Fig. 3C, 2 h) in CETPTg animals at early time points, as well as a similar trend for IL-1β, MCP-1, and IFNγ (Fig. 3D–F). Hepatic mRNA levels of IL-1β were unchanged in CETPTg mice compared with WT mice after 24 h (Fig. 3H). Taken together, these data suggest that the delayed clearance of purified LPS transiently alters the cytokine and chemokine burst when CETP is expressed. In order to determine whether CETP exerts its immunomodulatory effect when present in the plasma or in innate immune cells, we tested the ex-vivo cytokine/chemokine release from BMDMs from WT or CETPTg mice treated with increasing amounts of LPS in the presence of plasma from WT or CETPTg mice at different time points. As shown in Fig. 4, immune response was similar between macrophages from WT and CETPTg mice. However, cytokine production was significantly altered in the presence of plasma from CETPTg mice compared with plasma from WT mice. Indeed, in the presence of CETPTg plasma, when compared with WT plasma, IL-6 levels in culture media were decreased at 6 h with 1,000 ng/mL LPS (−42.2%, Fig. 4A); MCP-1 was increased at 24 h for all doses of LPS tested (+54.6%, +95.4%, and +46.7%, for 10, 100, and 1,000 ng/mL, respectively, Fig. 4B); IL-10 was increased when treated for 24 h with 10 mg/mL LPS (+53.3%, Fig. 4C); TNFα was increased at 2 h when treated with 10 and 100 ng/mL LPS (+34% and +37.3%, respectively, Fig. 4D); and IL-1β was increased at 6 h when treated with 100 and 1,000 ng/mL LPS (27.0-fold and 6.4-fold increases, respectively Fig. 4E) (*P* < 0.05 in all cases, Kruskal-Wallis followed by Dunn's multiple comparison test). To get more insight into the possible impact of CETP on mRNA levels of inflammatory mediators, macrophages were isolated from bone marrow from WT and CETPTg mice and submitted to LPS treatment (100 ng/mL, 6 h) in the presence of plasma from WT or CETPTg mice. As shown in supplemental Fig. S1, neither CETP expression in macrophages nor the presence of CETP in the plasma induced changes in cytokine mRNA levels.

**Fig. 5:**
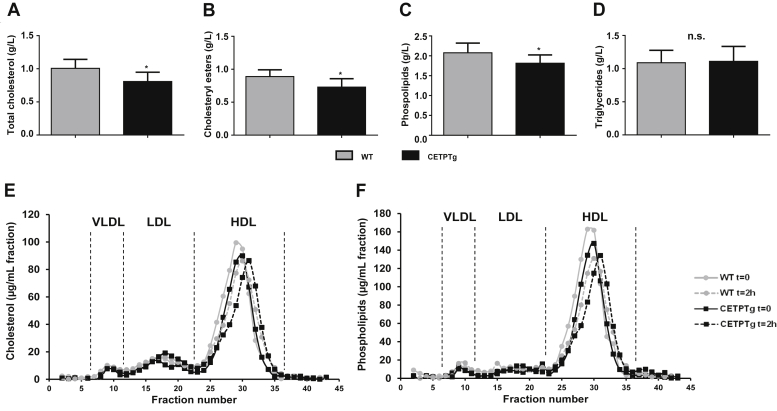
Plasma parameters and lipoprotein profile in mice after LPS iv injection. WT (n = 13) and CETPTg (n = 12) mice were iv injected with purified LPS from E. coli O55:B5 (15.0 mg/kg BW). (A) Total cholesterol, (B) esterified cholesterol, (C) phospholipids, and (D) triglycerides were measured in plasma 1 h after injection. Data are means ± SD. ∗*P* < 0.05, WT versus CETPTg; Mann-Whitney test. WT (n = 7) and CETPTg (n = 7) mice were iv injected with purified LPS from E. coli O55:B5 (15.0 mg/kg BW). Plasma samples were collected before (t = 0) and 2 h after injection and pooled by genotype for FPLC analysis. Fractions were collected to measure (E) total cholesterol and (F) phospholipids. n.s., nonsignificant.

Finally, in order to assess the impact of CETP on metabolic parameters and survival during endotoxemia, we challenged WT and CETPTg mice with a lethal dose of LPS (15 mg/kg) delivered iv. The presence of CETP in transgenic mice did not alter plasma cytokine levels two 2 h after LPS injection (supplemental Fig. S2). Leukocyte counts were identical at baseline and decreased after LPS injection, as expected from previous studies (20, 21, 22). The extent of this decrease was similar in WT and CETPTg mice (supplemental Fig. S3). However, total and esterified plasma cholesterol levels, which are known to be altered in CETPTg mice under basal conditions (23), remained lower at 1 h after LPS injection compared with WT counterparts (0.81 g/L ± 0.14 versus 1.01 g/L ± 0.14, and 0.73 g/L ± 0.13 versus 0.89 g/L ± 0.10, respectively; *P* < 0.05) (Fig. 5A, B). Plasma phospholipid levels were also found to be lower in CETPTg mice 1 h after LPS injection (1.81 g/L ± 0.21 vs. 2.08 g/L ± 0.24; *P* < 0.05) (Fig. 5C) while TG levels were similar in both groups (Fig. 5D). In order to get more insights into the impact of CETP expression on lipoprotein profile during severe endotoxemia, pooled plasmas obtained from WT and CETPTg mice at baseline and two 2 h after LPS injection (15 mg/kg) were passed through a size-exclusion chromatography device and cholesterol, phospholipid, TG, and protein concentrations were determined in individual fractions. As expected in mice, HDL appeared as the main carriers for cholesterol (more than 75%, Fig. 5E) and phospholipids (more than 80%, Fig. 5F) the in plasma, both in WT and in CETPTg mice. The HDL peak was smaller and shifted to a lower size range in CETPTg mice compared with WT mice at baseline and after LPS injection (Fig. 5E, F). In parallel, we also measured protein content in all FPLC fractions. At baseline, cholesterol-to-protein ratio in HDL was 15% lower in CETPTg mice compared with WT mice. LPS injection did not induce any other change in cholesterol-to-protein ratio in HDL from both genotypes, and an 11% reduction in this parameter was still observable in CETPTg mice. A similar trend could be observed in LDL (–17% at t = 0; –8% at t = 2 h) and in VLDL (–55% at t = 0; –41% at t = 2 h) when comparing CETPTg mice with WT mice. Phospholipid-to-protein ratios followed the same trend in HDL (–17% at t = 0; –9% at t = 2 h), LDL (–16% at t = 0; –31% at t = 2 h), and VLDL (39% at t = 0 h; –53% at t = 2 h) in CETPTg mice compared with WT mice. Regarding TG-rich lipoproteins (VLDL), CETPTg mice displayed a 48% drop in TG-to-protein ratio at baseline, and this trait was still observed two 2 h after LPS injection (−37%).

**Fig. 6:**
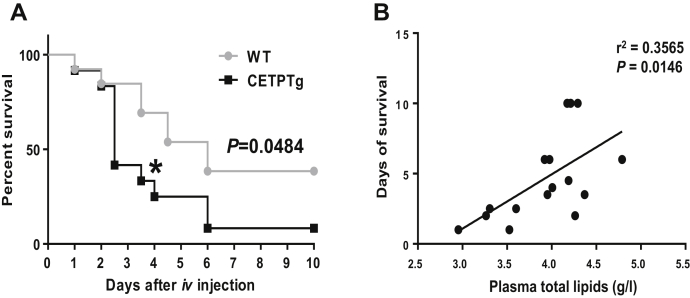
Survival after LPS injection. (A) Survival was monitored during 10 days on WT (n = 13) and CETPTg (n = 12) mice iv injected with purified LPS from E. coli O55:B5 (15 mg/kg BW). Data are percent survival measured at 12 h intervals. WT versus CETPTg; Kaplan-Meier method and log Rank test. ∗*P* < 0.05; Chi-squared test. (B) Relationship between days of survival and plasma total lipids (determined as the sum of plasma total cholesterol, triglycerides, and phospholipids) in a subset of WT (n = 7) and CETPTg mice (n = 9) was analyzed with Spearman correlation test.

After a 10-day period of follow-up, mortality rate was 50% higher in CETPTg mice than in WT counterparts (90% versus 60%, respectively; *P* < 0.05, log Rank test) (Fig. 6A). In order to evaluate whether higher mortality in mice might be linked to the amount of circulating lipids or cytokines, survival time after LPS injection was plotted against plasma concentrations of total lipids. A significant, positive correlation could be observed between survival time and plasma lipids (*r*^2^ = 0.3565; *P* = 0.0146, Spearman test, Fig. 6B).

### Effect of CETP on LPS detoxification and sepsis outcome in a CLP model

**Fig. 7:**
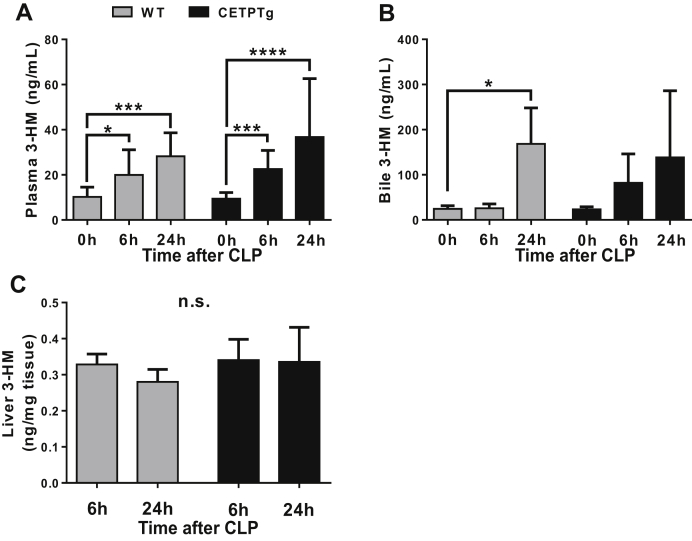
LPS distribution and excretion after CLP. WT (n = 4 to 8) and CETPTg (n = 4 to 8) mice were challenged with CLP. LPS concentration was obtained by direct quantitation of 3-HM in (A) plasma, (B) bile, and (C) liver using tandem mass spectroscopy. Data are means ± SD. ∗*P* < 0.05, ∗∗∗*P* < 0.001, ∗∗∗∗*P* < 0.0001; Kruskal-Wallis followed by Dunn's multiple comparisons test. n.s., nonsignificant.

As part of the outer membrane of gram-negative bacteria, most cases of endotoxemia do not consist of the presence of isolated LPS molecules only but rather in bacterial blebs or complex hydrophobic structures released during sepsis. In order to get more insights into the possible role of CETP on LPS detoxification and its consequences on sepsis outcome in a model of polymicrobial infection, we applied the CLP protocol to CETPTg and WT mice. In both WT and CETPTg mice, sepsis triggered a rapid and persistent elevation of LPS mass concentration in the plasma, with similar observations whether CETP was expressed or not (Fig. 7A). However, while WT mice displayed significant increase in biliary excretion of LPS after 24 h (168 ± 79 ng/mL at 24 h versus 24 ± 7 ng/mL at baseline, *P* < 0.05), the presence of CETP tended to limit LPS excretion with no significant accumulation in bile at 24 h compared with baseline (Fig. 7B). The LPS content of the liver was similar between 6 and 24 h of sepsis in WT and CETPTg mice (Fig. 7C).

Next, we checked the possible effect of CETP on metabolic parameters in our CLP model. Plasma lipid parameters were measured in WT and CETPTg mice at baseline (0 h, Table 1) and 6 and 24 h after application of the CLP procedure (Table 2). At baseline, and as expected (20), total and esterified cholesterol levels were lower in CETPTg mice as compared with WT mice (0.78 g/L ± 0.15 versus 0.92 g/L ± 0.20, and 0.46 g/L ± 0.09 versus 0.62 g/L ± 0.12, respectively; *P* < 0.05) (Table 1). CETPTg mice showed higher apoB-containing lipoproteins to HDL CE ratio as compared with WT mice (supplemental Fig. S4A). It is in line with expected outcome of CETP expression, and CETP activity remained stable along the sepsis episode (supplemental Fig S4B). Total cholesterol, esterified cholesterol, and phospholipid levels increased significantly between 6 and 24 h after CLP in wild-type mice only (Table 2). The same pattern was observed for ALT activity.

**Table 1:** Plasma parameters in mice at baseline

	Baseline	
	WT	CETPTg
Cholesterol (g/L)	0.92 ± 0.20	0.78 ± 0.15^a^
Esterified cholesterol (g/L)	0.59 ± 0.12	0.46 ± 0.09^a^
Triglycerides (g/L)	2.54 ± 1.00	2.79 ± 1.04
Phospholipids (g/L)	2.06 ± 0.41	1.91 ± 0.29
Glycaemia (mmol/L)	15.46 ± 2.63	15.64 ± 3.28
ALT (UI/L)	55.89 ± 40.46	45.35 ± 16.84

Lipid parameters, glycemia, and liver injury marker alanine aminotransferase (ALT) were measured at baseline in plasma of WT (n = 6 to 8) and CETPTg (n = 6 to 8) mice. Data are means ± SD.

a *P* < 0.05 versus WT, Mann-Whitney test.

**Table 2:** Plasma parameters in mice after CLP

	6 h after CLP		24 h after CLP	
	WT	CETPTg	WT	CETPTg
Cholesterol (g/L)	0.79 ± 0.14	0.81 ± 0.15	1.35 ± 0.22^a^	1.13 ± 0.24
Esterified cholesterol (g/L)	0.44 ± 0.09	0.45 ± 0.07	0.65 ± 0.15^b^	0.53 ± 0.14
Triglycerides (g/L)	1.20 ± 0.38	1.46 ± 0.46	1.51 ± 0.25	1.77 ± 0.65
Phospholipids (g/L)	1.64 ± 0.26	1.65 ± 0.24	2.52 ± 0.43^b^	2.06 ± 0.30
Glycaemia (mmol/L)	7.91 ± 1.85	7.17 ± 1.98	4.88 ± 0.87	5.30 ± 3.05
ALT (UI/L)	74.50 ± 25.50	82.56 ± 27.04	320.74 ± 82.94^b^	299.20 ± 47.92

WT (n = 6 to 8) and CETPTg (n = 6 to 8) mice were challenged with CLP. Lipid parameters, glycemia, and liver injury marker alanine aminotransferase (ALT) were measured in plasma 6 and 24 h after CLP. Data are means ± SD.

a *P* < 0.01 versus t6h; Kruskal-Wallis test with Dunn's multiple comparisons test.

b *P* < 0.05.

**Fig. 8:**
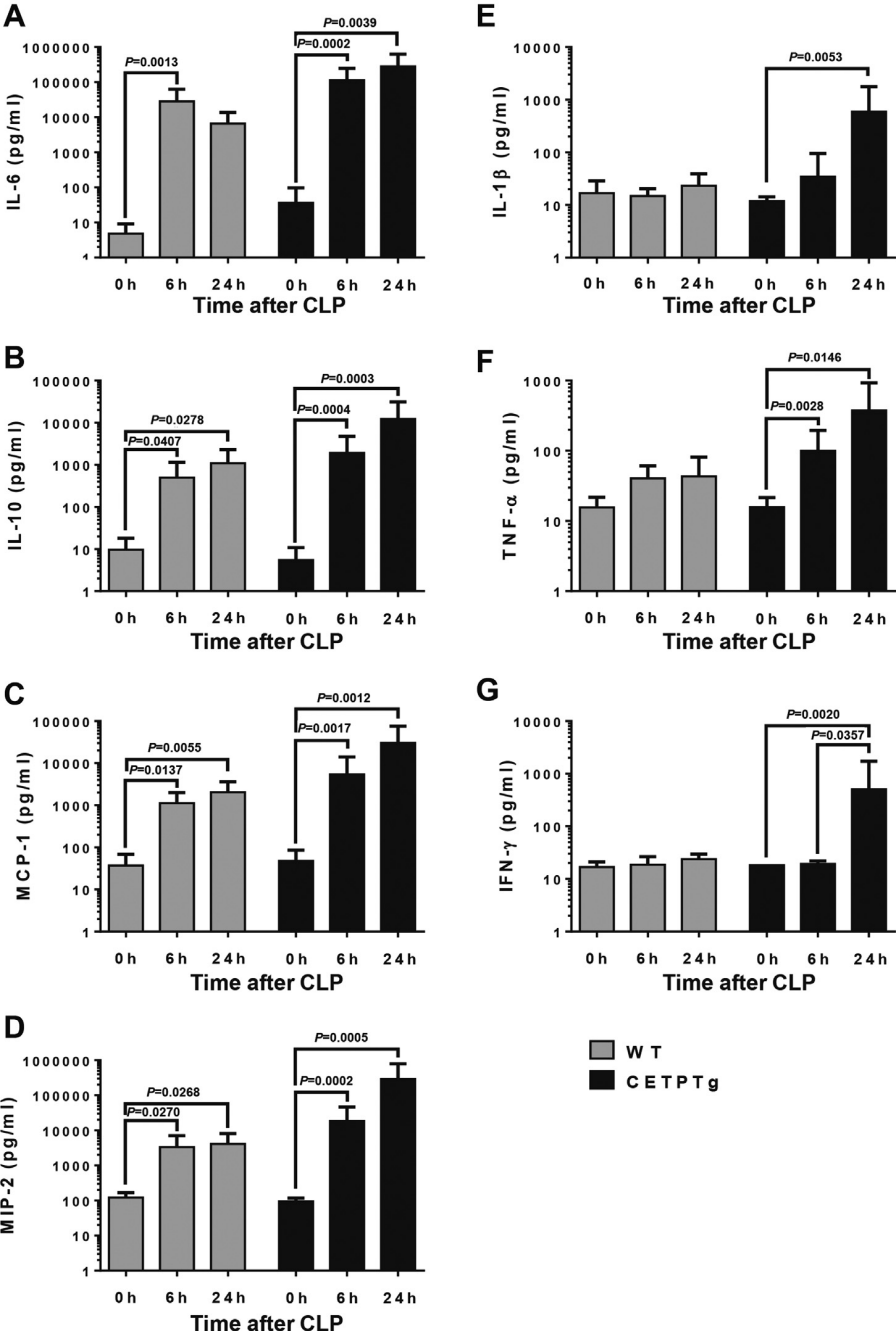
Plasma cytokine profile after CLP. WT (n = 6 to 8) and CETPTg (n = 6 to 8) mice were challenged with CLP. Plasma levels of IL-6 (A), IL-10 (B), MCP-1 (C), MIP-2 (D), IL-1β (E), TNFα (F), and IFNγ (G) were measured using Luminex assay. Data are means ± SD. *P*-values for significant differences (*P* < 0.05) are shown above corresponding brackets on each panel; Kruskal-Wallis followed by Dunn's multiple comparisons test.

The induction of sepsis originating from the peritoneal cavity induced the accumulation of leukocytes (WBC) in both WT and CETPT mice (supplemental Fig. S5A). This accumulation did not relate to alterations in lymphocyte counts (supplemental Fig. S5B) and only marginally to alterations in monocytes (supplemental Fig. S5C). Rather, leukocyte accumulation was mostly due to recruitment of PMNL, (supplemental Fig. S5D). Interestingly total WBC and PMNL progressively increased until 24 h in WT mice while they reached a peak at 6 h before going down at 24 h in CETPTg mice (supplemental Fig. S5A, D). In additional analyses, results were also expresses as fold-change compared with baseline in WT or CETPTg mice. In CETPTg mice, total leukocyte counts showed a significant 9.2-fold increase after 6 h (*P* = 0.0240), while monocytes were significantly by 10.8 and 6.2-fold at 6 and 24 h (*P* = 0.0030 and *P* = 0.0183, respectively), and PMNL were significantly increased by 18.2 and 13.4-fold (*P* = 0.0126 and *P* = 0.0392, respectively, Kruskal-Wallis test), while the changes were not statistically significant in WT mice. To investigate further whether CETP could affect systemic inflammatory response induced by sepsis, plasma levels of cytokines and chemokines were monitored over a 24 h period after CLP. Although the progressive rise in plasma levels of IL-6, IL-10, MCP-1, and MIP-2 after CLP was not affected by CETP expression (Fig. 8A–D), increases in plasma levels of IL-1β, TNFα, and IFNγ after 24 h were observed in CETPTg mice only (Fig. 8E–G).

**Fig. 9:**
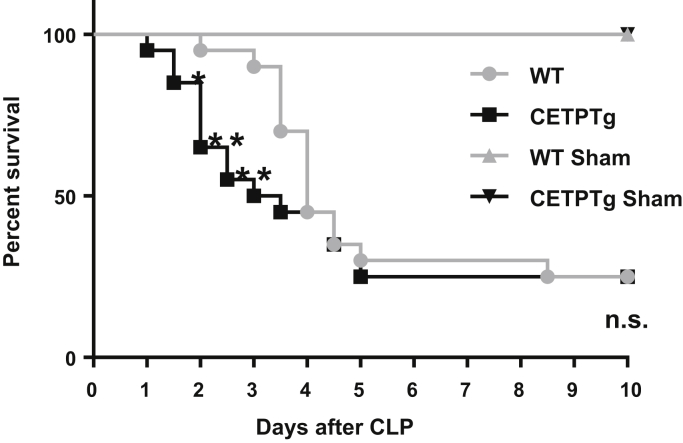
Survival after CLP. Survival was monitored during 10 days on WT (n = 20) and CETPTg (n = 20) mice challenged or not (Sham, n = 2) with CLP. Data are percent survival measured at 12 h intervals. WT versus CETPTg; Kaplan-Meier method and log Rank test. ∗*P* < 0.05, ∗∗*P* < 0.01; Chi-squared test.

Finally, the consequences of CETP expression on mouse survival after polymicrobial infection and endotoxemia were determined. A significantly higher rate of mortality could be observed in the early days in CETPTg than in WT mice, in particular with 50% versus 10% of mortality in CETPTg and WT mice after 3 days of sepsis, respectively (*P* < 0.05, χ^2^ test). Ten days after CLP, both WT and CETPTg groups displayed 75% of mortality (Fig. 9).

## Discussion

Although CETP belongs to the LT/LBP family, which includes members with well-described LPS transfer activities, the role of CETP in LPS transfer remains unclear. It is reported here that, unlike LBP, BPI, and PLTP, CETP has no intrinsic LPS transfer activity, with consistent in vitro and in vivo observations. Importantly, however, we could show that CETP expression was associated with a change in inflammatory burst and with earlier or higher mortality, both in polymicrobial infection and in acute endotoxemia models.

The first part of this work aimed at clarifying the role of CETP in LPS binding and transfer activity. Earlier in vitro works showed that CETP seems to be partially colocalized with LPS in the perinuclear region of macrophages (24), suggesting a possible interaction between these molecules; CETP was also found to enhance LPS binding to purified HDL and LDL at the expense of VLDL (25). However, these latter results could not be reproduced in vivo since we observed no increase in lipoprotein-bound LPS in CETPTg mice compared with wild-type animals in our model of polymicrobial infection. One of the drawbacks of the technique used in earlier in vitro studies comes from the use of purified LPS as a substrate for CETP (25) while circulating LPS is mostly found in host organisms as aggregates/micelles or as part of complex lipid structures derived from bacterial membranes during sepsis (26). In order to get more insight in the LPS transfer potency activity of CETP, we developed here a novel technique that allows us to monitor the net transfer of LPS from donors (HDL or VLDL) to acceptor lipoproteins. In addition, our transfer systems used total plasma as a source of endogenous lipid transfer proteins and lipoprotein acceptors, thus reflecting more accurately what may occur in vivo. Our technique could be validated by observing a significant decrease in LPS transfer activity in plasma from knockout mice lacking PLTP, i.e., one of the proteins known to bind and transfer LPS in vitro and in vivo, compared with the plasma from WT mice with naturally elevated plasma PLTP activity. Consistent observations were made using either HDL or VLDL as donors. In the presence of plasma from CETPTg mice, LPS transfer from HDL or VLDL to endogenous lipoproteins was not modified compared with the plasma from WT mice lacking CETP. The absence of LPS transfer activity of CETP contrasts with two other circulating members of the LT/LBP family, LBP and PLTP, which both bind and transfer LPS to lipoproteins. Taken together with the work of Clark and colleagues, who showed that CETP has a very low affinity for LPS (Kd ≥ 25 μM) (14), our data suggest that *1*) CETP has no direct interaction with LPS, and *2*) does not promote its transfer to and between lipoproteins. Therefore, the deleterious effect of CETP that we could observe in both models of endotoxemia and polymicrobial infection might relate to an indirect role of this protein on sepsis and endotoxemia, independently from LPS transfer.

Lipoproteins may play a pivotal role in the resistance against sepsis through different mechanisms. Firstly, HDLs harbor intrinsic anti-inflammatory properties by decreasing the expression of adhesion molecules in the vascular wall, the migration of immune cells, or the production of cytokines and chemokines (27). Secondly, while HDLs were shown to promote LPS transport back to the liver for biliary excretion (5, 28, 29), all lipoprotein classes are able to bind and inactivate LPS molecules that are present in the lymph and plasma (30). Due to its ability to exchange neutral lipids (CE and TG) between lipoproteins, CETP induces profound changes in the composition, functionality, and metabolic fate of lipoproteins, including decreased HDL cholesterol levels and HDL size in humans (31) and animal models (32, 33).

In accordance with a modulation of LPS clearance from the plasma, we could show that the presence of CETP could delay the clearance of endotoxins after a single iv injection of LPS. This observation could be explained by the propensity of CETP to modulate the ability of HDL to promote the clearance of circulating lipids by the liver (34, 35, 36). In addition, we could show a slight alteration of LPS biliary output in CETPTg mice compared with WT mice under CLP. The lack of apparent effect of CETP on the time course of plasma LPS levels in our sepsis model might relate to the peculiar kinetic behavior of LPS, which is progressively and constantly delivered into the circulation over at least 24 h after CLP (11, 37, 38, present study), contrarily to our endotoxemia model, which consists of an unique LPS bolus.

In clinics, a low cholesterol level at the day of admission is a poor prognostic of survival in patients suffering septic shock (39, 40) and is associated with more severe inflammatory burst and sepsis outcome in patients undergoing surgery under cardiopulmonary bypass (41). It indicates that the initial level of cholesterol is a critical parameter in sepsis outcome and inflammatory response. Recent work showed that a gain-of-function variant in *CETP* gene was associated with lower plasma HDL cholesterol levels, more pronounced proinflammatory cytokines release, and greater risk of sepsis-associated acute kidney injury (42). CETP-mediated HDL-C drop in septic patients was later shown to play a causal and deleterious role in survival from sepsis (43). Hence, basal changes in plasma total cholesterol and lipoprotein levels, as occurring in the presence of CETP, may significantly alter inflammation and sepsis outcome. Interestingly, under a chow diet, CETP expression in transgenic mice is also associated with decreased total cholesterol levels when compared with wild-type mice devoid of CETP (44, 45). The present study showed significant reductions in plasma levels of total and esterified cholesterol at the basal state and early time points in CETPTg mice compared with wild-type mice as well as significant decreases in plasma phospholipids in CETPTg mice compared with WT mice after LPS injection or during CLP. This consistent CETP-related decrease in plasma lipids in both models might relate to an enhancement of the remodeling of lipoproteins that is observed either at baseline or during inflammation (46). In support of this hypothesis, CETP was shown to favor PLTP-induced changes in HDL structure and composition in vitro (47). Accordingly, in the present study, FPLC analysis showed that the reduction in HDL size and lipid content in the presence of CETP tended to be further pronounced during endotoxemia. Several studies indicated that phospholipids reflect the total amount of lipoprotein surface as well as their capacity to bind and/or inactivate LPS while cholesterol and TGs are mostly linked with the content of lipoprotein core (48, 49, 50). Therefore we considered that the sum of total plasma lipids (cholesterol, TGs, and phospholipids) could reflect the overall capacity of plasma lipoproteins to counteract the deleterious effects of endotoxemia, and we could show that there was indeed a significant, positive correlation between total lipid values and survival rate in mice injected with LPS. In addition, we could also show ex vivo that plasma from CETPTg mice is able to modulate LPS-induced cytokine secretion from macrophages when compared with plasma from WT mice. The latter observation suggests that, beyond its influence on LPS kinetics, alterations of plasma profile in the presence of CETP directly impact the proinflammatory effect of LPS. Taken together, these data provide evidence that increased mortality and altered inflammatory response in CETPTg mice with endotoxemia or sepsis result from a general reduction in plasma lipid pool as a result from increased CETP-mediated lipoprotein remodeling and/or clearance.

Overall, whereas the present study comes in support of a significant role of CETP in modulating LPS clearance and LPS-mediated inflammation, its actual role and consequences must be evaluated and discussed in conjunction with other key players including immune cells, lipoproteins, and other LPS binding proteins. In addition, the outcome of CETP and lipoprotein actions might well depend on the time course of the inflammatory process. For instance, although HDLs are mostly considered as anti-inflammatory, they were shown to behave as proinflammatory factors in the early step of inflammation due to their ability to either unmask LBP or to deplete macrophage cholesterol (51, 52, 53). Interestingly, Blauw and colleagues have recently discussed the primacy of this early and transient proinflammatory effect of HDL to trigger a subsequent immune response required for sepsis resolution (54). As far as CETP is concerned, and beyond the intravascular LPS metabolism that was under the scope of the present study, it was found to modulate the expression of TLR-4 receptors (24). Finally, the importance of the IL-6/IL-10 balance was stressed in predicting the sepsis outcome and mortality (55). Because both IL-6 and IL-10 were found in the present study to be increased in CETPTg mice, the consequences of CETP on clinical outcome in relation with cytokine expression should be considered with caution and may not be restricted to sepsis and LPS clearance. Interestingly, total WBCs, mostly reflecting PMNL recruitment in the peritoneal cavity of mice subjected to CLP, showed a different pattern: while the recruitment was sustained until 24 h in WT mice, PMNL counts in CETPTg mice were transiently observed at 6 h only. These differences in the kinetics of PMNL recruitment in the peritoneal cavity might partly contribute to the differences in survival between CETPTg and WT mice after CLP.

In conclusion, our observations showed that CETP has no LPS binding or transfer capabilities in vitro and thus appears as an outlier in the LT/LBP family. Nevertheless, it is still able to influence the inflammatory response through its ability to alter lipoprotein levels, kinetics, and functionality. Although we could show that CETP is able to alter LPS transport and excretion in a model of isolated endotoxemia, the contribution of this phenomenon to the deleterious effect of CETP in our sepsis model seems to be modest. Rather, the lowering effect of CETP on lipid levels combined with altered intrinsic anti-inflammatory capacity of plasma lipoproteins might contribute to clinical outcome.

### Data availability

All data supporting the findings of the present study are contained in the manuscript or the supplemental file. Additional raw data are available upon request to corresponding author.
