# Supplementary material for: Human cholesteryl ester transfer protein lacks lipopolysaccharide transfer activity, but worsens inflammation and sepsis outcomes in mice
Source: J Lipid Res. 2020 Dec 15;62:100011. doi: 10.1194/jlr.RA120000704 (PMC7859855; doi:10.1194/jlr.RA120000704)
Supplement: Supplemental Figures S1 to S5 [file mmc1.pdf]

SUPPLEMENTAL FIGURES AND FIGURES LEGENDS

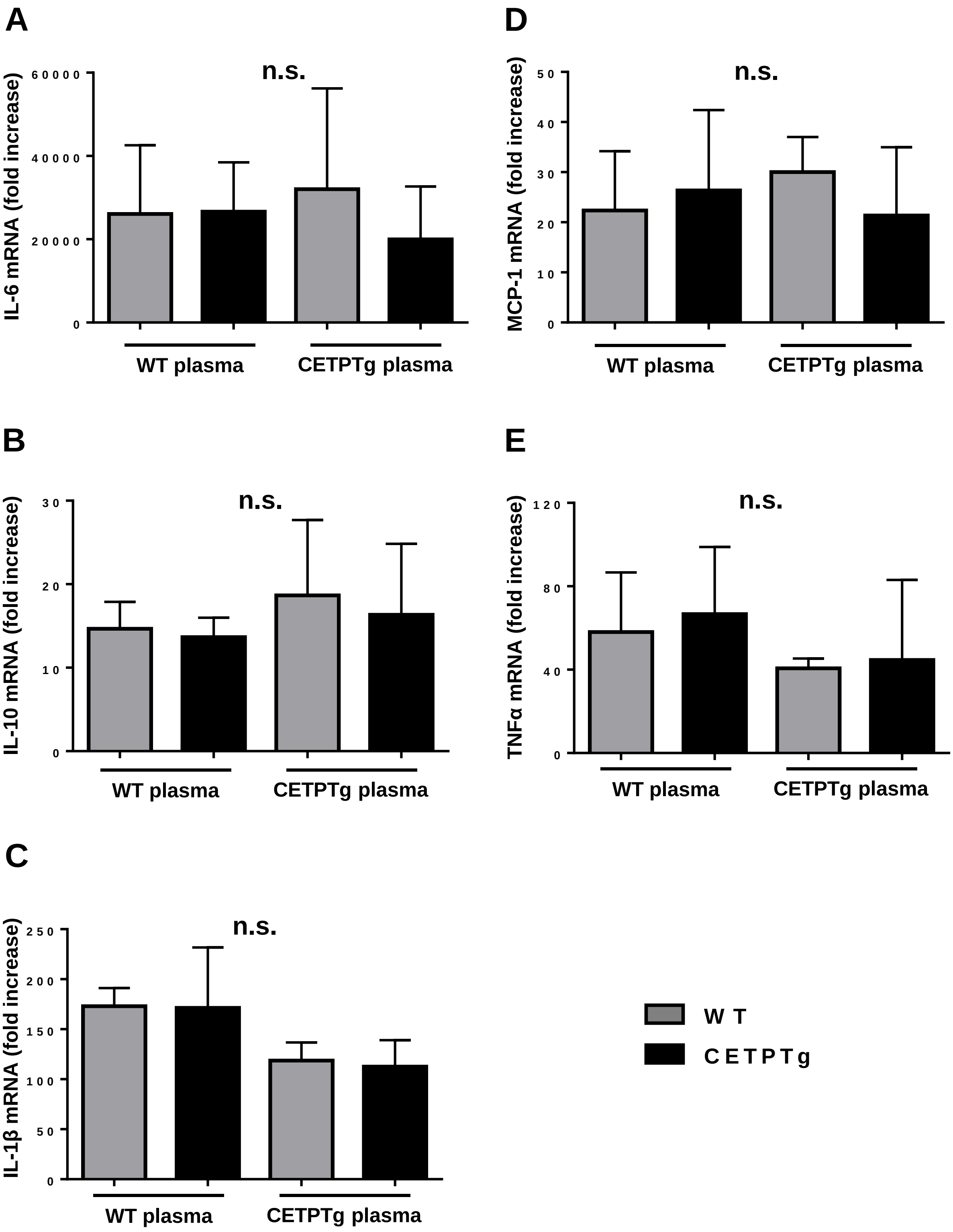

Supplemental Figure S1. Macrophage response to LPS in vitro

Bone-marrow-derived macrophages (BMDM) were isolated from WT (grey bars, n=3) and CETPTg mice (black bars, n=3) and cultured as described under Materials and Methods. LPS treatment (0.1µg/mL) was performed in the presence of serum from either WT (left bars) or CETPTg mice (right bars). BMDM response to LPS was assessed after 6h by measuring mRNA levels of IL-6 (A), IL-10 (B), IL-1β (C), MCP-1 (D) and TNFα by quantitative PCR. Results were expressed as fold increase compared with untreated cells. Data are means ± SD and were compared by using Kruskal-Wallis followed by Dunn's multiple comparisons test. n.s.: non significant.

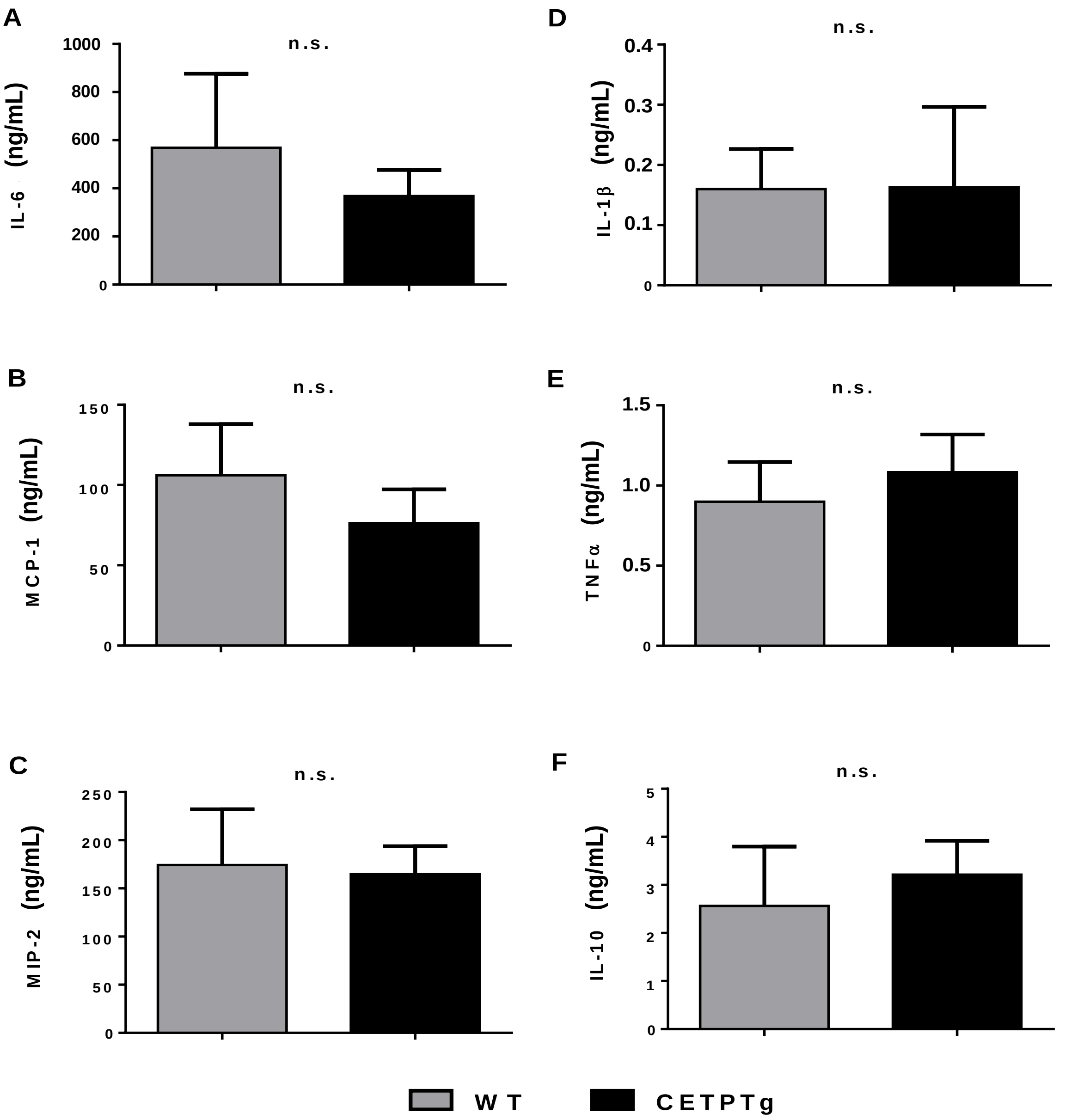

Supplemental Figure S2. Plasma cytokine profile after high dose LPS injection.

WT (n=7) and CETPTg (n=7) mice were *iv* injected with purified LPS from E. coli O55:B5 (15.0 mg/kg BW). Plasma levels of IL-6 (**A**), MCP-1 (**B**), MIP-2 (**C**), IL-1β (**D**), TNFα (**E**), and IL-10 (**F**) were measured using Luminex assay 2h after injection. Data are means ± SD. WT vs. CETPTg; Mann-Whitney test. n.s.: non significant.

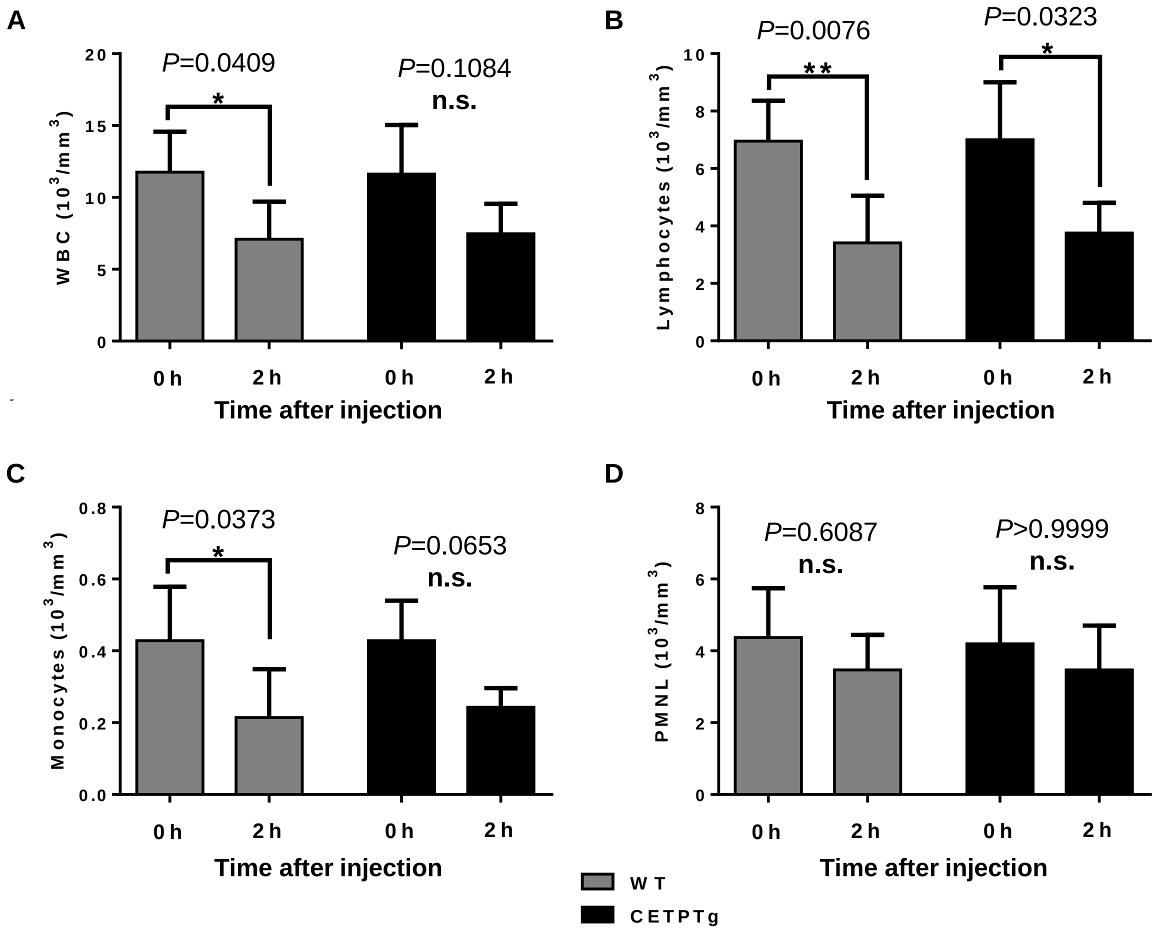

Supplemental Figure S3. Blood leukocytes after LPS injection.

WT (n=7) and CETPTg (n=7) mice were *iv* injected with purified LPS from E. coli O55:B5 (15.0 mg/kg BW). Blood counts of (**A**) WBC, (**B**) lymphocytes, (**C**) monocytes, and (**D**) PMNL were measured using an automated hematology analyzer. Data are means ± SD. *P*-values for significant differences are shown above corresponding brackets on each panel. **P*<0.05, ***P*<0.01; Kruskal-Wallis followed by Dunn's multiple comparisons test. n.s.: non significant. WBC : White Blood Cells. PMNL : Polymorphonuclear Leukocytes.

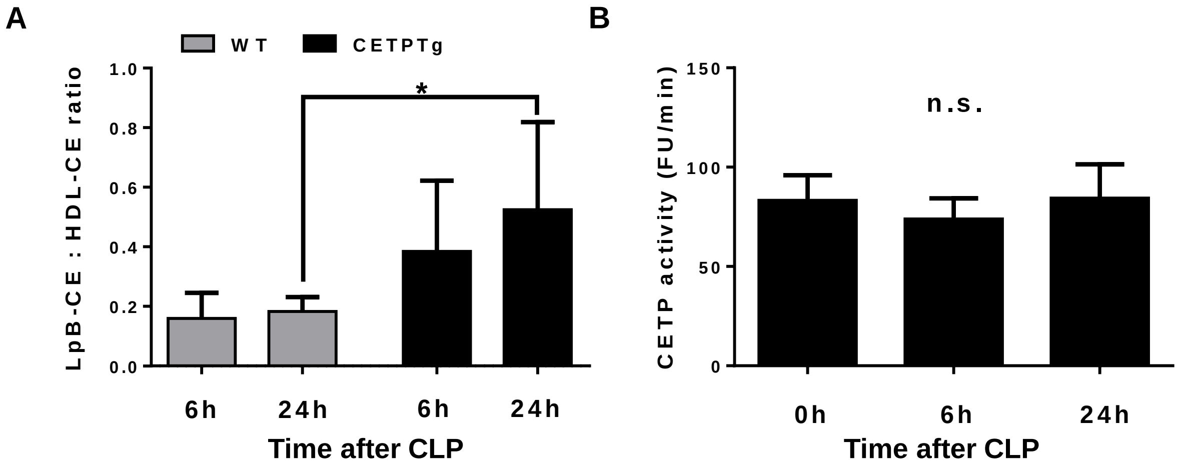

Supplemental Figure S4. CETP activity and cholesteryl ester distribution after CLP.

WT (n=6 to 8) and CETPTg (n=6 to 8) mice were challenged with CLP. (**A**) Cholesteryl ester concentrations were measured in Apo-B containing lipoproteins (LpB-CE) and HDL (HDL-CE) fractions separated from plasma by gradient ultracentrifugation. (**B**) CETP activity in plasma from CETPTg animals was assessed with a fluorescence-based commercial kit. Data are means ± SD. **P*<0.05; Kruskal-Wallis followed by Dunn's multiple comparisons test. n.s.: non significant.

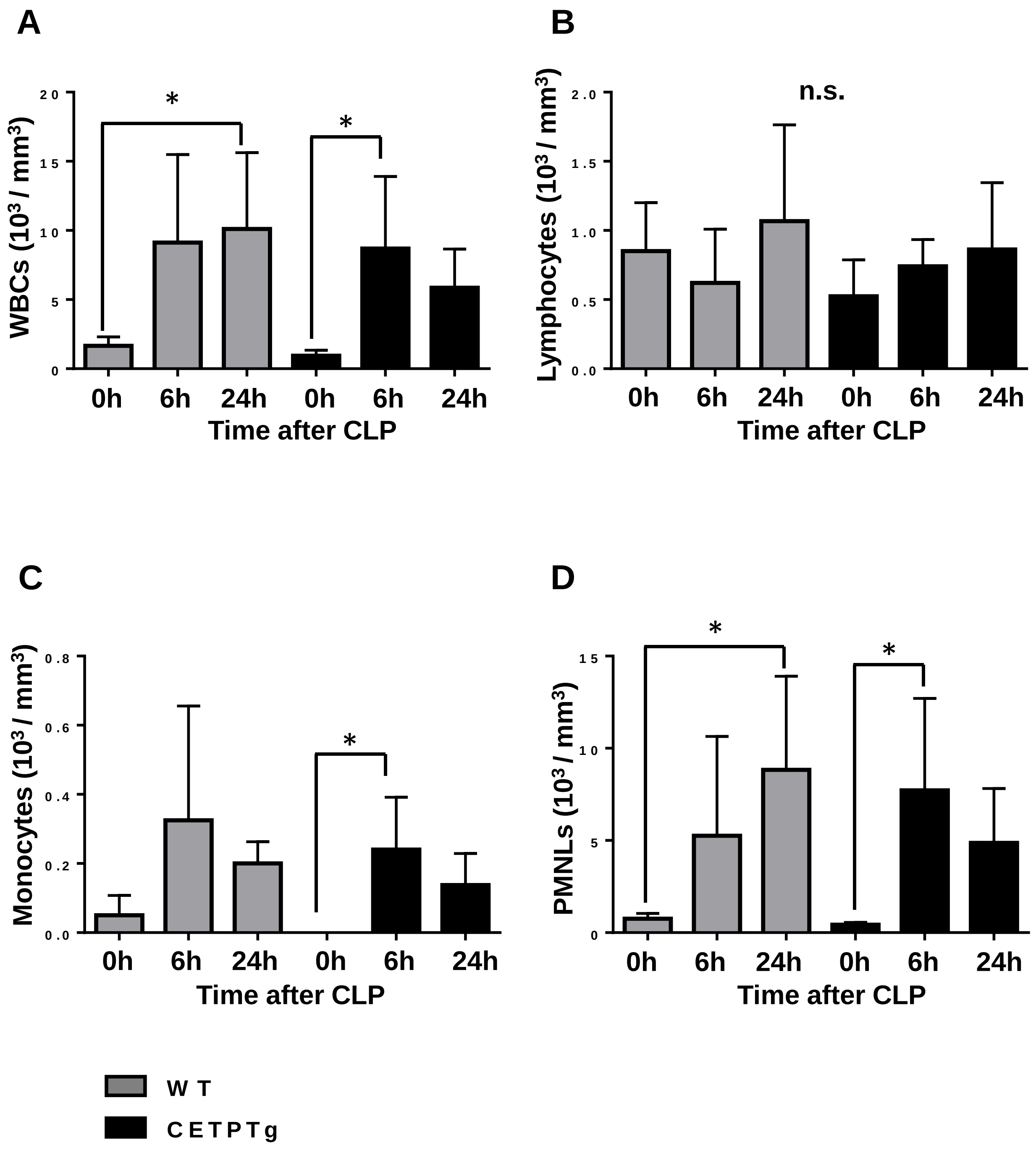

Supplemental Figure S5. Peritoneal leukocytes after CLP.

WT and CETPTg mice (n=4-7) underwent the CLP procedure and euthanized at 0, 6 and 24 hours for peritoneal lavage. Peritoneal counts of (**A**) WBC, (**B**) lymphocytes, (**C**) monocytes, and (**D**) PMNL were measured using an automated hematology analyzer. Data are means ± SD. **P*<0.05; Kruskal-Wallis followed by Dunn's multiple comparisons test. n.s.: non significant. WBC : White Blood Cells. PMNL : Polymorphonuclear Leukocytes.
